# Neuregulin 1 Gene (*NRG1*). A Potentially New Targetable Alteration for the Treatment of Lung Cancer

**DOI:** 10.3390/cancers13205038

**Published:** 2021-10-09

**Authors:** Daniel Rosas, Luis E. Raez, Alessandro Russo, Christian Rolfo

**Affiliations:** 1 The Internal Medicine Department, University of Texas Health Science Center at San Antonio, San Antonio, TX 78229, USA; 2 Thoracic Oncology Program, Memorial Cancer Institute/Memorial Health Care System, Florida International University (FIU), Miami, FL 33021, USA; lraez@mhs.net; 3 Medical Oncology Unit, A.O Papardo, 981258 Messina, Italy; alessandrorusso@aopapardo.it; 4 Clinical Research and Center for Thoracic Oncology, The Tisch Cancer Institute, Mount Sinai Health System & Icahn School of Medicine at Mount Sinai, New York, NY 10029, USA; christian.rolfo@mssm.edu

**Keywords:** *NRG1* fusion, lung cancer, resistance to therapy, target therapy

## Abstract

**Simple Summary:**

Treatment in oncology has and will keep evolving into an agnostic approach where therapies are guided more towards the identification and targeting of genetic abnormalities and less by organ of origin of the cancer, as has been done for decades. With every genetic abnormality being identified as a target, the pharmaceutical development of medications targeting these genes has grown, leading to better survival rates, quality of life and a bigger interest in finding new targets. Lung cancer is one of the best examples where targetable genetic abnormalities have led to substantial survival differences compared to patients undergoing empirical conventional chemotherapy. Translocations in the neuregulin 1 gene (*NRG1*) are one of many gene fusions that are becoming clinically significant, and it has the potential to become a targetable gene with ongoing clinical trials already in Europe and the US. This review aims to portray the importance and latest developments regarding this new fusion in lung cancer treatment.

**Abstract:**

Oncogenic gene fusions are hybrid genes that result from structural DNA rearrangements, leading to unregulated cell proliferation by different mechanisms in a wide variety of cancer. This has led to the development of directed therapies to antagonize a variety of mechanisms that lead to cell growth or proliferation. Multiple oncogene fusions are currently targeted in lung cancer treatment, such as those involving *ALK, RET, NTRK* and *ROS1* among many others. Neuregulin (NRG) gene fusion has been described in the development of normal tissue as well as in a variety of diseases, such as schizophrenia, Hirschsprung’s disease, atrial fibrillation and, most recently, the development of various types of solid tumors, such as renal, gastric, pancreatic, breast, colorectal and, more recently, lung cancer. The mechanism for this is that the *NRG1* chimeric ligand leads to aberrant activation of *ERBB2* signaling via PI3K-AKT and MAPK cellular cascades, leading to cell division and proliferation. Details regarding the incidence of these gene rearrangements are lacking. Limited case reports and case series have evaluated their clinicopathologic features and prognostic significance in the lung cancer population. Taking this into account, *NRG1* could become a targetable alteration in selected patients. This review highlights how the knowledge of new molecular mechanisms of *NRG1* fusion may help in gaining new insights into the molecular status of lung cancer patients and unveil a novel targetable molecular marker.

## 1. Introduction

Diagnostic and therapeutic resources in medical oncology are and will continue to evolve into a more individualized approach. The presence or absence of specific genetic abnormalities will guide treatment and also help as markers for prognosis, medication response and survival. In the last decade, multiple pharmaceutical agents have been approved as targeted therapies by the FDA. Some examples of targetable gene abnormalities are those involving *EGFR, ALK, BRAF, ROS, RET, KRASg12c, HER2, PI3K, MET exon 14, NTRK, PD1* and, more recently, *IDH1/2* and *FGFR*. This has led to questions, such as what other molecular markers are responsible for oncogenic development, but also which ones can be targeted and which ones can be detected, not only with issue but also with blood work such as liquid biopsies. Oncogenic gene fusions are hybrid genes that result from structural DNA rearrangements, leading to deregulated activity.

**Figure 1:**
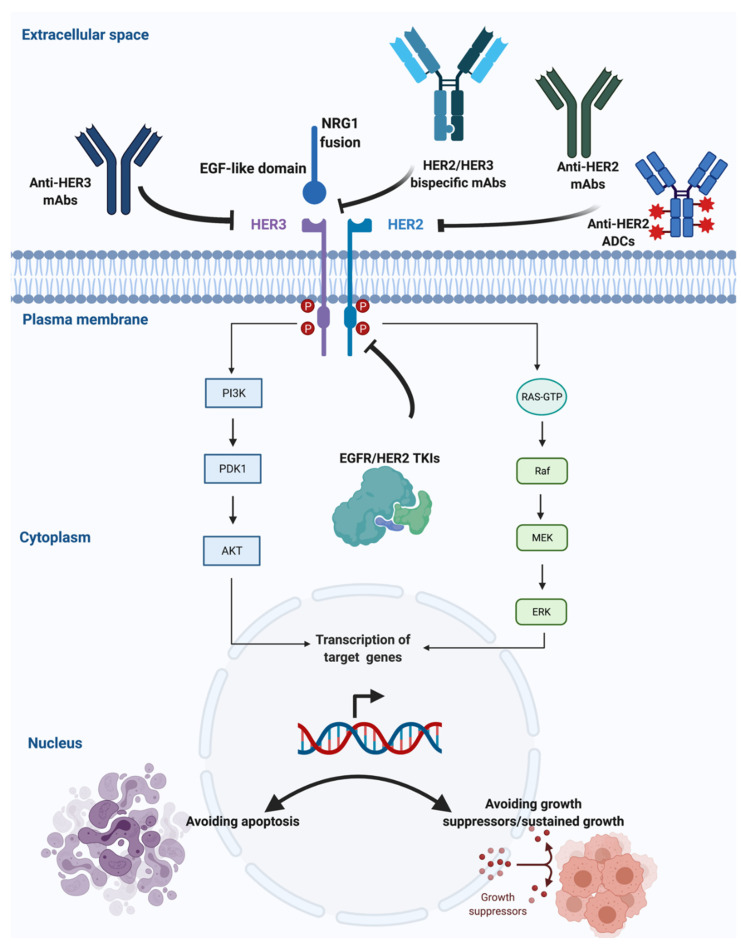
Targeting *NRG1* rearrangements in solid tumors (Credit: created with BioRender.com, accessed on 4 July 2021).

The *NRG1* gene is located in chromosome 8 in region 8p12. This gene encodes the growth factor neuregulin 1 (*NRG1*). *NRG1* contains an epidermal growth factor (EGF)-like domain, which binds to human tyrosine kinases of the ErbB/HER receptor group, specifically ERBB3 and ERBB4, leading to the activation of ErbB-mediated downstream signaling pathways that translate into cell growth. This has led to the development of targeted therapies to *NRG1* that are currently underway (Figure 1) [1,2,3,4].

*NRG1* can create fusions with other genes, and the most common fusion partners identified in patients with lung cancer include *SLC3A2, SDC4, RBPMS, WRN, VAMP2, ATP1B1, ROCK1, RALGAPA1, TNC, MDK, DIP2B, MRPL13, DPYSL2, PARP8* and *ITGB1*. In samples with other types of cancer, not including lung, *POMK* (colorectal cancer, CRC), *APP* (pancreatic ductal adenocarcinoma, PDAC), *CDH6* (PDAC), *ATP1B1* (cholangiocarcinoma and PDAC) and *CLU* (ovarian cancer) were the most common fusions found [5,6].

## 2. Early Studies in *NRG1*

There are reports of tumors expressing concomitant *NRG1* rearrangements with known protooncogenes such as *ALK* or *KRAS*. Medical oncologists could potentially use this as an advantage for treatment, since some tyrosine kinase inhibitors (TKI) are non-selective to not just one receptor or mutation but to multiple, taking advantage of those tumors with multiple targetable mutations [7,8,9].

Regarding non-neoplastic conditions, *NRG1* expression has been identified as an adaptive response to tissue alteration. The systems that this has been described are the cardiac, gastrointestinal tissues, as well as the nervous system. In the specific example of heart failure, when cardiomyocytes are injures or overloaded, *NRG1* expression increases, leading to fibroblast and macrophage activation. This has led to studies in which *NRG1* is administered to patients with heart failure, improving cardiac function in different models, and is currently being researched in other pathologies such as atrial fibrillation as well as other cardiac diseases, such as Hirschsprung’s disease [10,11,12].

Regarding the nervous system, the presence or absence of the *NRG1* gene has shown a relationship with Alzheimer’s disease. A study by Mouton-Liger et al. showed that a high *NRG1* expression in cerebrospinal fluid (CSF) shows a negative correlation with cognition in Alzheimer’s disease patients. Other studies show a positive correlation with cognition in patients with a diagnosis of schizophrenia and even a protective correlation for cortical stroke treatment [13,14,15,16].

## 3. *NRG1* and Early Reports in Cancer

*NRG1* gene fusions have been identified in multiple types of cancers. Jonna et al. profiled 21,858 tumor specimens over a 3-year time spam and found the incidence to be 0.2%. The greatest incidence was in non-small-cell lung cancer (NSCLC). Other tumor types harboring an *NRG1* fusion included PDAC, CRC, gastrointestinal stromal tumors (GISTs), squamous cell carcinomas (SCCs), breast, cholangiocarcinoma, thyroid, renal cell carcinoma, bladder, ovarian, neuroendocrine and sarcoma and are clinically actionable oncogenic drivers [17,18,19,20,21]. In another cohort of patients, Drilon et al. reported 17,485 patients with a variety of advanced solid tumors, where *NRG1* rearrangements were detected in 0.14% (3/2079) of NSCLC cases, specifically lung adenocarcinomas, 0.13% (1/791) of pancreatic adenocarcinomas and 0.04% (1/2703) of patients with ER+/HER2-positive breast cancer. Of note, they describe how in patients with wild-type *KRAS* lung cancer, *NRG1* fusions were detected in 11% of patients (4 of 36) [22]. Regarding epidemiology, Fernandez-Cuesta et al. found that *NRG1* rearrangements are more common in those that have never smoked. By screening 102 lung adenocarcinomas negative for known oncogenic alterations, they found that *NRG1* was present in 4 out of 15 of the invasive mucinous adenocarcinoma (IMA) subtype [18].

Kim et al. report the treatment of two patients with lung IMA *NRG1*+ that were treated with lumretuzumab, a monoclonal anti-ERBB3 antibody, in combination with erlotinib during a clinical trial. Both patients were treated in a setting of more than three lines of therapy failure. At least sixteen weeks of progression-free survival (PFS) were achieved without any unacceptable toxicity. Given that IMA is a rare but aggressive disease, this small case series show how other options for treatment should be further studied, such as targeting HER2 for patients with *NRG1* rearrangements [23]. Howarth et al. describe a complex mechanism of *NRG1* alterations, where some mutations can lead to increase cell proliferation and evasion of apoptosis but on the contrary, some *NRG1* fusion proteins can lead to cell death. The authors theorize that not only the upregulation of this pathway but also its downregulation can lead to cell proliferation. This theory encourages more research in the signal pathway to determine if not only inhibition of this pathway but maybe agonism can lead to tumor regression. Whether or not this is the explanation, because many *NRG1* rearrangements seem to be inactivating, the correct identification of activating fusions may require care [24].

ERBB2-positive breast cancer is treated with directed therapy as the standard of care. If patients develop resistance to HER2-targeted therapies, Yang et al. theorize that *NRG1* expression could be responsible for HER2 resistance, specifically to trastuzumab, making this gene abnormality even more attractive to research since it could potentially represent not only another targetable receptor but a modifiable one with which to avoid resistance to anti-HER2 therapies [25]. Specifically in breast cancer, some authors, such as Prentice et al., report that *NRG1* rearrangements can represent a poor prognosis factor [26].

The most common treatment approach to patients with lung cancer is to receive chemotherapy with or without surgery and radiation. Patients that relapse or become resistant to multiple modalities receive molecular studies such as next-generation sequencing (NGS) to determine the next best approach to treatment if an actionable mutation is present. Hegde et al. hypothesize that chemotherapy may induce *NRG1* expression in tumor cells, making them resistant to its cytotoxic effects and leading to chemotherapy resistance [27]. Cadranel et al. published a case series of six patients harboring *NRG1* gene fusions, five with LMA and one with CRC, and all were treated with afatinib. From the five lung cancer patients, four had a partial response (PR) and one had stable disease (SD). The CRC patient had stable disease. Of note, 100% of patients were treated not as a first-line treatment, and most were in the setting of failing multiple lines of treatment. A conclusion by this case series is that *NRG1* inhibitors can be an option for patients who have already had undergone multiples lines of treatment [28]. Jones et al. published a case series of 47 patient with pancreatic ductal adenocarcinoma from which three (67%) were found to have *NGR1* rearrangements and received afatinib. These three patients were identified as wild-type *KRAS* by whole-genome sequencing. All wild-type *KRAS* tumors were positive for gene fusions involving the ERBB3 ligand *NRG1*. Two of three patients with *NRG1* fusion-positive tumors were treated with afatinib and demonstrated a significant and rapid response while on therapy. One of these patients had a family history of gastrointestinal cancers (colon and gastric), and another patient had a family history of prostate and colon cancer. All this contributes to the growing amount of evidence that not only could *NRG1* represent a targetable alteration, but also that its presence increases the risk of different types of tumor; it could, potentially, be used as a genetic assessment in liquid biopsies. These authors point out that the mechanisms of resistance to *NRG1*-targeting agents could be potentially explained by the upregulation of *NRG1* as well as parallel pathway activation, as seen in HER2-positive breast cancer models and ALK-positive lung cancer [29]. Yung et al. evaluated the presence of *NRG1* in 502 gastric cancer samples and found that 28.1 % (141 patients) were expressors. *NRG1* overexpression was significantly associated with aggressive features, including infiltrative tumor growth, lymphovascular and neural invasion, a high pathologic stage and poor prognosis, but it was not associated with the presence of EBV, MSI or HER2 status. These results suggest that *NRG1* overexpression may predict poor clinical outcomes and that targeting *NRG1* represents a therapeutic opportunity in gastric cancer [30]. Duruisseaux et al. reported a case series of 25 patients from France with a diagnosis of IMA. A driver oncogene was identified in 14/25 IMAs, namely 12 *KRAS* mutations (48%), 1 *ROS1* rearrangement (4%) and 1 *ALK* rearrangement (4%). The detection of *NRG1* rearrangements was conducted in 11 pan-negative IMA. One *NRG1* rearrangement which was a 61-year-old non-smoking woman of Vietnamese ethnicity, and was the sole patient of Asian ethnicity in the cohort. This patient had a history of breast cancer treated with neoadjuvant chemotherapy, radiation and surgery, so there are many factors that could potentially lead to *NRG1* rearrangements. The authors conclude two main points. First, that *NRG1* FISH detection should be considered in patients with pan-negative IMA, and second, these results might suggest that *NRG1* abnormalities are more frequent in IMA in patients of Asian descent, but with the acknowledgement that the cohort of patient was small [31]. Jones et al. published a two-case-series treatment report published in the Annals of Oncology where one patient had lung adenocarcinoma and one patient had cholangiocarcinoma. Both patients had different *NRG1* rearrangements, but both were treated with the TKI afatinib. Both displayed a significant and durable response to treatment [32].

## 4. Recent NRG Studies Reported

Results of the eNRGy1 Global Multicenter Registry were published recently. This is the largest retrospective study evaluating the clinic-pathological characteristics and outcomes of lung cancer patients harboring *NRG1* rearrangements, providing useful information regarding testing methods and responses to convectional therapies as well as afatinib. *NRG1* gene fusions were more common in those that had never smoked (57%), in non-metastatic patients (71%) and in the IMA subtype (57%). The use of RNA sequencing was associated with higher detection rates when compared to DNA sequencing. In addition, patients harboring *NRG1* rearrangements tended to exhibit low response rates and short PFS when treated with platinum-based or taxane-based chemotherapy (ORRs 13% and 14%, respectively; median PFS 5.8 and 4.0 months, respectively) and with chemo-immunotherapy (ORR 0% and PFS 3.3 months) or immunotherapy alone (ORR 20% and PFS 3.6 months). Among 110 patients, 20 patients received afatinib as a treatment for metastatic disease, with encouraging signals of activity (ORR 25% and PFS 2.8 months), regardless of fusion partner [33]. Laskin et al. report a 19-case series where multiple types of tumors were included and it was seen that the minimum number of months of response was three and the maximum was more than 36 months. This authors also comment that *NRG1* could potentially not only be a targetable alteration but also a potential good prognostic factor, although available data are conflicting [5]. Pan et al. reported that 115 surgical specimens who underwent lung cancer resection were analyzed and showed that negative expression of *NRG1* was associated with overall survival (OS) and a lower probability of recurrence. Of note, more than 50% of tumors samples had no concomitant other gene alterations besides *NRG1* [34]. In another study, Shin et al. evaluated the presence of *NRG1* rearrangements in 59 patients with IMA, showing that the concomitant presence of other driver mutations is detectable in a significant proportion of cases (10/16 *NRG1* fusion-positive cases had concurrent *KRAS* mutations and two additional cases harbored an *NRAS* mutation and an *ALK* fusion, respectively). Interestingly, the presence of an *SLC3A2-NRG1* rearrangement was associated with poor OS [35].

## 5. Discussion: Where Are We Going with *NRG1*

Ongoing and future research will determine if and when *NRG1* can become a target for agnostic treatment and shift standard-of-care treatment options, just as other targeted therapies have. *NRG1* fusions are present in multiple cancer types and in a relative high proportion of lung cancer, specifically IMA, which is one of the most aggressive types of lung cancer. Although these gene fusions are relatively uncommon in most cancer types, they are detectable and targetable. Other *NRG1*-positive tumor types include pancreatic, gallbladder cancer, renal cell carcinoma, bladder cancer, ovarian cancer, breast cancer, neuroendocrine tumor, sarcoma and CRC, showing how an actionable medication could benefit a large group of patients with a large variety of tumors. Currently, there are multiple clinical trials ongoing attempting to either target or amplify *NRG1* for different conditions such as heart failure and multiple neoplasia. Multiple phase I, II and III trials are underway, assessing how using the understanding of *NRG1* directly can impact treatment considerations and even prognostic models (NCT03388593, NCT01214096, NCT01439893 and NCT01439789) [36,37,38]. A phase II clinical trial aims to investigate the efficacy of the pan-ERBB inhibitor afatinib in advanced-stage *NRG1*-rearranged malignancies across all tumor entities following progression in standard therapy (NCT04410653) [39]. An open-label, single-arm, phase IV clinical study was designed to evaluate the efficacy of afatinib in the treatment of *NRG1*-fused locally advanced/metastatic NSCLC and explore the clinical factors that may predict the effectiveness of treatment (NCT04814056) [40]. Phase II clinical trials are evaluating seribantumab, a novel monoclonal antibody against HER3, which binds HER3 and inhibits *NRG1*-dependent activation and HER2 dimerization. This study is in patient with recurrent, locally advanced or metastatic solid tumors, including metastatic pancreatic cancer harboring *NRG1* gene fusions (NCT04790695, NCT04383210) [41,42]. An open-label phase II trial for patients with various stages of NSCLC and other solid tumors is recruiting patients with NSCLC (EGFR exon 20 insertion, HER2-activating mutations) and other solid tumors with *NRG1/ERBB* gene fusions to be treated with tarloxotinib bromide (NCT03805841) [43]. Another phase I/II study is studying single-agent zenocutuzumab (MCLA-128) in patients with solid tumors, including NSCLC and pancreatic cancer, harboring an *NRG1* fusion. Zenocutuzumab is a full-length IgG1 bispecific antibody targeting HER2 and HER3 (NCT02912949) [44]. Recently, the preliminary results of the phase I/II global clinical trial eNRGy in advanced solid tumors harboring *NRG1* rearrangements were presented. In total, 47 patients were included (25 NSCLC, 12 PDAC and 10 solid tumors with different histologies). In patients with PDAC, an impressive 42% ORR was reported with an additional 50% of patients achieving SD. Responses were seen regardless of tumor histology (ORR in the overall cohort was 29%) and fusion partners. Treatment was well-tolerated with most of the adverse events of grade 1–2 [45]. Based on these results, the FDA granted fast-track designation to zenocutuzumab.

It is the authors’ opinion that the mentioned studies highlight the potential clinical importance that *NRG1* can have, but acknowledge the limited data and the rareness of its presence in the cancer population, being somewhat specific to lung cancer patients. With broader next-generation sequencing testing of tumor samples, this gene abnormality will become more prevalent and subsequently so will its research and potential clinical significance. One question the authors pose is whether the effect of agonisms, antagonisms and mixed effects on this class of receptors could be compared to the ones seen in medications such as selective estrogen receptor modulators (SERMs) in the case of breast cancer, where they are an agonist of estrogen in some tissues and an antagonist in others. SERMs have been widely used for the treatment of breast cancer, where they have shown clinical benefits when they agonize and antagonize the same class of receptors in different tissues.

*NRG1* also opens the door to debate as to whether other diagnostic tools such as liquid biopsy can be used to detect this mutation and target it. Given the now widely used next-generation sequencing (NGS) diagnostic tool, which detects such mutations in tumor tissues and in blood, it becomes a matter of whether *NRG1* can be a clinically targeted mutation. With this in mind, the authors also state that this diagnostic tool is not 100% sensitive or specific. This has the pitfall of creating higher rates of false-positive results of *NRG1* presence that could potentially translate into targeted therapies that could lead to adverse effects for this patient population.

## 6. Conclusions

In conclusion, *NRG1* mutations defined a unique molecular subgroup for further research. The *NRG1* gene was originally studied for its role in the development and damage response pathway of cardiac, nervous and gastrointestinal tissues. Currently it is regarded as an oncogene of increasing importance, with potential targeted-therapy implications. Currently, we have valuable information on the targetable potential of this mutation that will continue to grow and incorporate higher numbers of patients and a broader inclusion of other tumor types besides lung cancer. This will inevitably lead to further studies of its mechanisms of resistance for this combination regimen. The development of new drugs for rare diseases is challenging, but the evaluation of drugs already approved for other indications is a pragmatic option. Options for personalized lung cancer therapy will be increased with the help of multiplex diagnosis systems able to detect multiple druggable gene fusions. It is essential to pursue promising therapies that may provide meaningful clinical benefits for individuals whose tumors harbor *NRG1* fusions.

## References

[1] Muscarella L.A., Rossi A. (2017). NRG1: A cinderella fusion in lung cancer?. Lung Cancer Manag..

[2] Trombetta D., Rossi A., Fabrizio F.P., Sparaneo A., Graziano P., Fazio V.M., Muscarella L.A. (2017). NRG1-ErbB Lost in Translation: A New Paradigm for Lung Cancer?. Curr. Med. Chem..

[3] Fernandez-Cuesta L., Thomas R.K. (2015). Molecular Pathways: Targeting NRG1 Fusions in Lung Cancer. Clin. Cancer Res..

[4] Dimou A., Camidge D.R. (2019). Detection of NRG1 Fusions in Solid Tumors: Rare Gold?. Clin. Cancer Res..

[5] Laskin J., Liu S., Tolba K., Heining C., Schlenk R., Cheema P., Cadranel J., Jones M., Drilon A., Cseh A. (2020). NRG1 fusion-driven tumors: Biology, detection, and the therapeutic role of afatinib and other ErbB-targeting agents. Ann. Oncol..

[6] Shin D.H., Jo J.Y., Han J.-Y. (2018). Dual Targeting of ERBB2/ERBB3 for the Treatment of SLC3A2-NRG1–Mediated Lung Cancer. Mol. Cancer Ther..

[7] Muscarella L.A., Trombetta D., Fabrizio F.P., Scarpa A., Fazio V.M., Maiello E., Rossi A., Graziano P. (2017). ALK and NRG1 Fusions Coexist in a Patient with Signet Ring Cell Lung Adenocarcinoma. J. Thorac. Oncol..

[8] Wang Y., Ning Z., Zhou X., Yang Z., Tang H., Xu M., Wang X., Zhao J., Bai Y. (2018). Neuregulin1 acts as a suppressor in human lung adenocarcinoma via AKT and ERK1/2 pathway. J. Thorac. Dis..

[9] Xia D., Le L.P., Iafrate A.J., Lennerz J. (2016). KIF13B-NRG1 Gene Fusion and KRAS Amplification in a Case of Natural Progression of Lung Cancer. Int. J. Surg. Pathol..

[10] Dugaucquier L., Feyen E., Mateiu L., Bruyns T.A.M.J., De Keulenaer G.W., Segers V.F.M. (2020). The role of endothelial autocrine NRG1/ERBB4 signaling in cardiac remodeling. Am. J. Physiol. Circ. Physiol..

[11] Gunadi, Budi N.Y.P., Sethi R., Fauzi A.R., Kalim A.S., Indrawan T., Iskandar K., Makhmudi A., Adrianto I., San L.P. (2018). NRG1 variant effects in patients with Hirschsprung disease. BMC Pediatr..

[12] Zhou X., Wang Z., Huang B., Yuan S., Sheng X., Yu L., Meng G., Wang Y., Po S.S., Jiang H. (2018). Regulation of the NRG1/ErbB4 Pathway in the Intrinsic Cardiac Nervous System Is a Potential Treatment for Atrial Fibrillation. Front. Physiol..

[13] Mouton-Liger F., Dumurgier J., Cognat E., Hourregue C., Zetterberg H., Vanderstichele H., Vanmechelen E., Bouaziz-Amar E., Blennow K., Hugon J. (2020). CSF levels of the BACE1 substrate NRG1 correlate with cognition in Alzheimer’s disease. Alzheimer’s Res. Ther..

[14] Munafò M.R., Thiselton D.L., Clark T., Flint J., Munaf M.R. (2006). Association of the NRG1 gene and schizophrenia: A meta-analysis. Mol. Psychiatry.

[15] Rajasekaran A., Shivakumar V., Kalmady S.V., Parlikar R., Chhabra H., Prabhu A., Subbanna M., Venugopal D., Amaresha A.C., Agarwal S.M. (2020). Impact of NRG1 HapICE gene variants on digit ratio and dermatoglyphic measures in schizophrenia. Asian J. Psychiatry.

[16] Navarro-González C., Huerga-Gómez A., Fazzari P. (2019). Nrg1 Intracellular Signaling Is Neuroprotective upon Stroke. Oxidative Med. Cell. Longev..

[17] Jonna S., Feldman R., Swensen J., Gatalica Z., Korn W.M., Borghaei H., Ma P.C., Nieva J.J., Spira A.I., Vanderwalde A.M. (2019). Detection of NRG1 Gene Fusions in Solid Tumors. Clin. Cancer Res..

[18] Fernandez-Cuesta L., Plenker D., Osada H., Sun R., Menon R., Leenders F., Ortiz-Cuaran S., Peifer M., Bos M., Daßler J. (2014). CD74–NRG1 Fusions in Lung Adenocarcinoma. Cancer Discov..

[19] Halama N., Haberkorn U. (2020). The Unmet Needs of the Diagnosis, Staging, and Treatment of Gastrointestinal Tumors. Semin. Nucl. Med..

[20] Hegde G.V., De La Cruz C., Giltnane J.M., Crocker L., Venkatanarayan A., Schaefer G., Dunlap D., Hoeck J., Piskol R., Gnad F. (2019). NRG1 is a critical regulator of differentiation in TP63-driven squamous cell carcinoma. eLife.

[21] Zhang T., Qu N., Sun G., Zhang L., Wang Y., Mu X., Wei W.-J., Wang Y., Ji Q., Zhu Y. (2018). NRG1 regulates redox homeostasis via NRF2 in papillary thyroid cancer. Int. J. Oncol..

[22] Drilon A., Somwar R., Mangatt B.P., Edgren H., Desmeules P., Ruusulehto A., Smith R.S., Delasos L., Vojnic M., Plodkowski A.J. (2018). Response to ERBB3-Directed Targeted Therapy in NRG1-Rearranged Cancers. Cancer Discov..

[23] Kim H.S., Han J.-Y., Shin D.H., Lim K.Y., Lee G.K., Kim J.Y., Jacob W., Ceppi M., Weisser M., James I. (2018). EGFR and HER3 signaling blockade in invasive mucinous lung adenocarcinoma harboring an NRG1 fusion. Lung Cancer.

[24] Howarth K.D., Mirza T., Cooke S.L., Chin S.-F., Pole J.C., Turro E., Eldridge M.D., Garcia R.M., Rueda O.M., Boursnell C. (2021). NRG1 fusions in breast cancer. Breast Cancer Res..

[25] Yang L., Li Y., Shen E., Cao F., Li L., Li X., Wang X., Kariminia S., Chang B., Li H. (2017). NRG1-dependent activation of HER3 induces primary resistance to trastuzumab in HER2-overexpressing breast cancer cells. Int. J. Oncol..

[26] Prentice L.M., Shadeo A., Lestou V.S., A Miller M., Deleeuw R.J., Makretsov N., Turbin D., Brown L., Macpherson N., Yorida E. (2005). NRG1 gene rearrangements in clinical breast cancer: Identification of an adjacent novel amplicon associated with poor prognosis. Oncogene.

[27] Hegde G.V., de la Cruz C.C., Chiu C., Alag N., Schaefer G., Crocker L., Ross S., Goldenberg D., Merchant M., Tien J. (2013). Blocking NRG1 and Other Ligand-Mediated Her4 Signaling Enhances the Magnitude and Duration of the Chemotherapeutic Response of Non-Small Cell Lung Cancer. Sci. Transl. Med..

[28] Cadranel J., Liu S.V., Duruisseaux M., Branden E., Goto Y., Weinberg B.A., Heining C., Schlenk R.F., Cheema P., Jones M.R. (2021). Therapeutic Potential of Afatinib in NRG1 Fusion-Driven Solid Tumors: A Case Series. Oncolology.

[29] Jones M.R., Williamson L.M., Topham J.T., Lee M.K.C., Goytain A., Ho J., E Denroche R., Jang G.-H., Pleasance E.D., Shen Y. (2019). NRG1 gene fusions are recurrent, clinically actionable gene rearrangements in KRAS wild-type pancreatic ductal adenocarcinoma. Clin. Cancer Res..

[30] Yun S., Koh J., Nam S.K., Park J.O., Lee S.M., Lee K., Lee K.S., Ahn S.-H., Park D.J., Kim H.-H. (2018). Clinical significance of overexpression of NRG1 and its receptors, HER3 and HER4, in gastric cancer patients. Gastric Cancer.

[31] Duruisseaux M., McLeer-Florin A., Antoine M., Alavizadeh S., Poulot V., Lacave R., Rabbe N., Cadranel J., Wislez M. (2016). NRG1 fusion in a French cohort of invasive mucinous lung adenocarcinoma. Cancer Med..

[32] Jones M., Lim H., Shen Y., Pleasance E., Ch’Ng C., Reisle C., Leelakumari S., Zhao C., Yip S., Ho J. (2017). Successful targeting of the NRG1 pathway indicates novel treatment strategy for metastatic cancer. Ann. Oncol..

[33] Drilon A., Duruisseaux M., Han J.-Y., Ito M., Falcon C., Yang S.-R., Murciano-Goroff Y.R., Chen H., Okada M., Molina M.A. (2021). Clinicopathologic Features and Response to Therapy of NRG1 Fusion–Driven Lung Cancers: The eNRGy1 Global Multicenter Registry. J. Clin. Oncol..

[34] Pan B., Wang R., Zhang J., Chen H., Huang Y., Garfield D. (2015). HGF and NRG1 protein expression are not poor prognostic markers in surgically resected lung adenocarcinoma. Oncotarget Ther..

[35] Shin D.H., Lee D., Hong D.W., Hong S.H., Hwang J.-A., Lee B.I., You H.J., Lee G.K., Kim I.-H., Lee Y.-S. (2016). Oncogenic function and clinical implications of SLC3A2-NRG1 fusion in invasive mucinous adenocarcinoma of the lung. Oncotarget.

[36] Zensun Sci. & Tech. Co., Ltd. A Multi-Center, Randomized, Double-Blined, Placebo Parallel Controlled Phase III Clinical Trial to Evaluate the Effect of Injectable Neucardin on the Mortality of Subjects with Chronic Systolic Heart Failure on Standard HF Therapy–clinicaltrials.gov; 2021. https://clinicaltrials.gov/ct2/show/NCT03388593.

[37] Zensun Sci. & Tech. Co., Ltd. A Multi-Center, Randomized, Double-Blind, Placebo Parallel Controlled, Standard Therapy Based Phase III Clinical Trial to Evaluate the Efficacy and Safety of Recombinant Human Neuregulin-1 for Subcutaneous Administration in Patients with Chronic Systolic Heart Failure–clinicaltrials.gov; 2017. https://clinicaltrials.gov/ct2/show/NCT01214096.

[38] Zensun Sci. & Tech. Co., Ltd. A Phase III, Multi-Center, Randomized, Double-Blind, Based on Standard Therapy, Placebo-Controlled Study of the Efficacy/Safety of Recombinant Human Neuregulin-1β in Patients With Chronic Systolic Heart Failure–clinicaltrials.gov; 2017. https://clinicaltrials.gov/ct2/show/NCT01439893.

[39] German Cancer Research Center Afatinib in Advanced NRG1-Rearranged Malignancies: The NCT/DKTK PMO-1604 Phase-II Trial–clinicaltrials.gov; 2021. https://clinicaltrials.gov/ct2/show/NCT04410653.

[40] Shun L. An Open-Labeled, Single-Arm Clinical Study to Evaluate the Efficacy of Afatinib in Treatment of Locally Advanced or Metastatic Non-Small Cell Lung Cancer with NRG1-Fusion–clinicaltrials.gov; 2021. https://clinicaltrials.gov/ct2/show/NCT04814056.

[41] Ottawa Hospital Research Institute Single Patient Protocol for an NRG1 Fusion Positive Metastatic Pancreatic Cancer Patient Using Seribantumab–clinicaltrials.gov; 2021. https://clinicaltrials.gov/ct2/show/NCT04790695.

[42] Elevation Oncology CRESTONE: A Phase 2 Study of Seribantumab in Adult Patients with Neuregulin-1 (NRG1) Fusion Positive Locally Advanced or Metastatic Solid Tumors–clinicaltrials.gov; 2021. https://clinicaltrials.gov/ct2/show/NCT04383210.

[43] Rain Therapeutics Inc. Phase 2 Study–Evaluate the Clinical Activity of Tarloxotinib in Patients with Non-Small Cell Lung Cancer That Harbors an EGFR Exon 20 Insertion or HER2-Activating Mutation and Other Advanced Solid Tumors with NRG1/ERBB Family Gene Fusions–clinicaltrials.gov; 2020. https://clinicaltrials.gov/ct2/show/NCT03805841.

[44] Merus N.V. A Phase I/II Study of MCLA-128, a Full Length IgG1 Bispecific Antibody Targeting HER2 and HER3, in Patients with Solid Tumors–clinicaltrials.gov; 2021. https://clinicaltrials.gov/ct2/show/NCT02912949.

[45] Schram A.M., O’Reilly E.M., O’Kane G.M., Goto K., Kim D.-W., Neuzillet C., Martin-Romano P., Duruisseaux M., Nagasaka M., Rodon J. (2021). Efficacy and safety of zenocutuzumab in advanced pancreas cancer and other solid tumors harboring NRG1 fusions. J. Clin. Oncol..

